# Training variability and threshold density: a conceptual comparison of East African and Norwegian endurance training systems

**DOI:** 10.3389/fphys.2026.1878492

**Published:** 2026-06-17

**Authors:** Gerasimos V. Grivas, Walaa Jumah Alkasasbeh, Ioannis Trigonis, Konstantinos Karakatsanis, Adam Tawfiq Amawi

**Affiliations:** 1 Physical Education and Sports, Division of Humanities and Political Sciences, Hellenic Naval Academy, Piraeus, Athens, Greece; 2 Department of Physical Education, School of Sports Sciences, The University of Jordan, Amman, Jordan; 3 Department of Physical Education and Sport Science, Democritus University of Thrace, Komotini, Greece; 4 Department of Movement Sciences and Sports Training, School of Sports Sciences, The University of Jordan, Amman, Jordan

**Keywords:** distance running, endurance training, lactate threshold, threshold density, training intensity distribution, training variability

## Abstract

East African and Norwegian endurance training systems have both produced world-class distance runners, despite substantial differences in how training is organized, regulated, and executed. East African training is commonly characterized by group-based execution, variable pacing, fartlek-style sessions, and internally regulated intensity, often within altitude-associated environments. In contrast, the Norwegian model emphasizes threshold-density training, lactate-guided intensity control, structured interval formats, and repeated moderate-intensity sessions embedded within a high volume of low-intensity work. The purpose of this narrative review was to compare these two endurance training systems, examine their underlying physiological characteristics, and propose a conceptual framework describing distinct pathways to elite endurance performance. The available literature suggests that variability-driven training may favor adaptability, recruitment diversity, and tolerance to pace fluctuations, whereas threshold-density training, embedded within a high volume of low-intensity work, may promote metabolic efficiency, lactate regulation, and steady-state durability. Although these systems differ markedly in training organization, both appear capable of supporting world-class endurance performance. These observations suggest that elite endurance performance does not depend on a single optimal training model but may emerge through different organizational routes to physiological adaptation and performance development.

## 1 Introduction

Endurance running performance is determined by complex interactions among physiological capacity, training structure, environmental exposure, and long-term athlete development (Joyner and Coyle, 2008; Seiler, 2010). Traditional endurance training models have emphasized training volume and intensity distribution as primary determinants of performance, with polarized and pyramidal intensity distributions commonly reported in elite endurance athletes (Seiler and Kjerland, 2006; Seiler, 2010; Stöggl and Sperlich, 2015; Sun et al., 2025). However, emerging evidence suggests that different high-performance systems may achieve elite outcomes through distinct training organization, intensity control strategies, and long-term athlete development approaches (Wilber and Pitsiladis, 2012; Tønnessen et al., 2014; Sandbakk and Holmberg, 2017).

East African runners, particularly from Kenya and Ethiopia, have dominated middle- and long-distance running for several decades (Grivas et al., 2024). Their success has been associated with a combination of high training volume, altitude exposure, group-based training, and moderate-intensity variability, often including fartlek-style workouts and internally regulated pacing (Saltin et al., 1995; Larsen, 2003; Wilber and Pitsiladis, 2012). These training environments emphasize collective training dynamics, perceived effort, and progressive aerobic development rather than strict physiological monitoring (Wilber and Pitsiladis, 2012; Santos-Concejero et al., 2015).

In contrast, the Norwegian endurance training model has recently gained attention due to the success of athletes such as Jakob Ingebrigtsen and other elite Norwegian endurance athletes, as well as world-class performers in cross-country skiing and other Olympic endurance sports (Sandbakk and Holmberg, 2017; Kelemen et al., 2024). This approach emphasizes frequent threshold training, lactate-guided intensity control, and carefully structured interval sessions characterized by high accumulated volume and controlled execution. The model typically combines a large proportion of low-intensity training with repeated moderate-intensity interval sessions, aiming to maximize aerobic adaptation while minimizing excessive fatigue and high-intensity overload (Tønnessen et al., 2014, Tønnessen et al., 2024; Sandbakk and Holmberg, 2017; Sandbakk et al., 2025).

**Figure 1:**
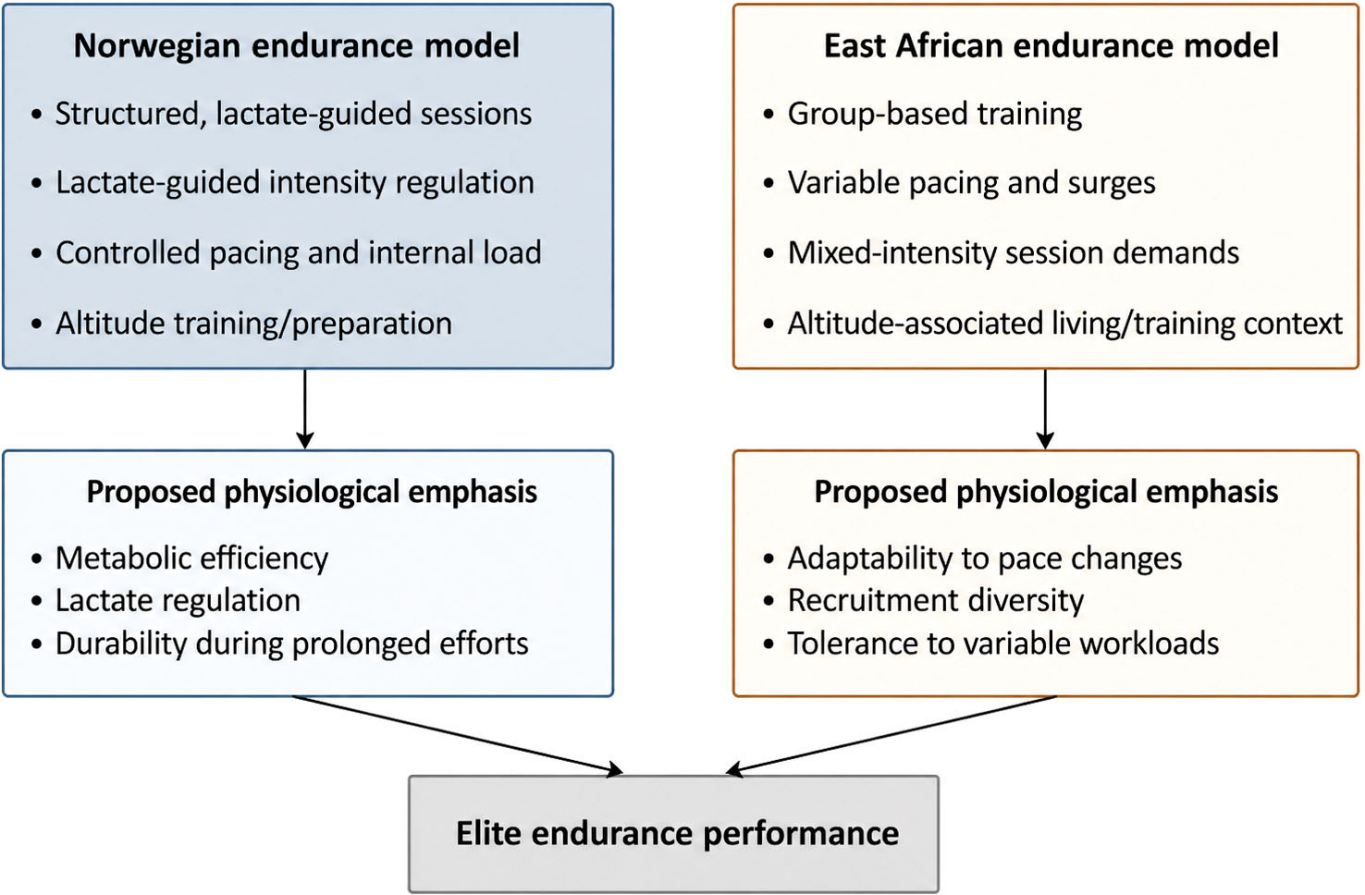
Conceptual comparison of selected characteristics of variability-driven (East African) and threshold-density (Norwegian) endurance training models, their proposed physiological emphases, and their relationship to elite endurance performance.

Despite their contrasting structures, both training systems have produced world-class endurance athletes, suggesting that distinct training organizations may represent alternative pathways toward elite endurance performance. Understanding these pathways is important for both scientific interpretation and practical coaching applications. Identifying shared principles and distinctive elements may provide useful insights into how different endurance training systems organize training load and physiological adaptation. These insights may help coaches adapt training strategies across different athlete populations and training environments. Therefore, the purpose of this paper is to compare East African and Norwegian endurance training models, examine their underlying physiological characteristics, and propose a conceptual framework describing two distinct pathways to elite endurance performance. The paper also discusses how selected elements of these models may inform training practice in different athletic contexts (**Figure 1**).

## 2 Methodology

This narrative review synthesizes peer-reviewed evidence comparing endurance training organization in East African and Norwegian distance running systems and their potential physiological implications. Literature was identified through targeted searches in PubMed, Scopus, and Web of Science using combinations of terms such as “East African runners”, “Kenyan training”, “Ethiopian runners”, “Ugandan runners”, “Norwegian training”, “lactate-guided training”, “training variability”, “fartlek”, “training intensity distribution”, and “elite distance runners”. Priority was given to observational studies of East African athletes, descriptive analyses of Norwegian endurance training, and investigations examining structured intensity control, pacing variability, and training organization. Because this study is a narrative rather than a systematic review, no formal inclusion or exclusion criteria were applied. Instead, studies were selected based on relevance to endurance training organization, methodological quality, and contribution to mechanistic understanding of physiological adaptations. Athlete performance level referenced in the included studies was interpreted according to the framework proposed by McKay et al. (2022), categorizing runners as recreationally active (Tier 1), trained/developmental (Tier 2), highly trained/national level (Tier 3), elite/international level (Tier 4), or world class (Tier 5). Both descriptive field observations and applied physiological investigations were considered appropriate, given the limited availability of controlled experimental studies in highly trained and elite endurance populations. This qualitative synthesis was used to compare training organization characteristics, identify complementary physiological adaptation pathways, and develop a conceptual framework describing how variability-driven and threshold-density training approaches may contribute to elite endurance performance.

## 3 The East African endurance training model

The dominance of East African runners in middle- and long-distance running has been widely documented, particularly among athletes from Kenya and Ethiopia, with Ugandan runners emerging more recently as major contributors to international success (Grivas et al., 2024). Their performance has been attributed to a multifactorial combination of environmental exposure, long-term development, and distinctive training characteristics (Saltin et al., 1995; Wilber and Pitsiladis, 2012; Casado et al., 2022; Haugen et al., 2022).

Training practices among East African runners are typically characterized by high weekly running volume, altitude exposure, and group-based training environments (Grivas et al., 2024). Athletes frequently train collectively, with pacing emerging organically within groups rather than being externally prescribed. This group-based structure promotes internally regulated intensity and variability in training stimulus (Wilber and Pitsiladis, 2012; Santos-Concejero et al., 2015).

An important feature of East African training is the ability to sustain relatively high-intensity workloads even at moderate altitude. Previous studies have reported that East African runners accumulate a greater proportion of training volume at intensities exceeding 80% of VO_2max_ compared with non-African athletes, despite similar low-intensity training volume (Coetzer et al., 1993). In addition, elite Kenyan runners frequently perform interval training at or above vVO_2max_, including sessions such as repeated 200 m intervals at approximately 120% vVO_2max_ and longer interval workouts at threshold intensity (Billat et al., 2003). Tempo running and sustained efforts near the lactate threshold (LT) also represent key components of East African training. Continuous tempo runs lasting 45–70 min and long aerobic intervals near LT velocity are commonly performed, often combined with fartlek-style sessions and hill running (Grivas et al., 2024). These workouts are typically conducted on varied terrain and emphasize progressive aerobic development and fatigue resistance rather than strict physiological monitoring (Larsen, 2003; Wilber and Pitsiladis, 2012; Casado et al., 2022).

Another characteristic of East African runners is their capacity to maintain cerebral oxygenation during maximal self-paced exercise, even at altitude, which may contribute to sustained high-intensity performance. This physiological resilience, combined with variability in training intensity and group-based pacing dynamics, may enhance endurance adaptations and fatigue resistance (Santos-Concejero et al., 2015).

Collectively, these characteristics indicate that East African training emphasizes variability-driven aerobic stress, internally regulated intensity, and flexible training organization. This ecological training environment may represent a distinct pathway toward elite endurance performance.

### 3.1 Training volume, frequency, and group-based dynamics

East African runners typically accumulate high weekly training volumes distributed across multiple daily sessions. Distance runners from Kenya and Ethiopia commonly perform between 100 and 160 km per week, with some athletes exceeding this range depending on training phase and competitive level (Saltin et al., 1995; Billat et al., 2003). This training volume is generally distributed across 10–14 sessions per week and often includes two training sessions per day, allowing athletes to accumulate substantial aerobic work while maintaining manageable fatigue levels (Wilber and Pitsiladis, 2012; Haugen et al., 2022).

A large proportion of this training is performed at low-to-moderate intensity, enabling athletes to tolerate high training frequency and maintain consistency across the weekly microcycle (Casado et al., 2022). Morning sessions typically consist of easy aerobic running, whereas afternoon sessions may include fartlek workouts, tempo runs, or interval training (Larsen, 2003). Long runs also represent a central component of East African training. These sessions are commonly performed once per week and typically last between 90 and 120 min, although longer durations may be observed in marathon-focused athletes (Wilber and Pitsiladis, 2012). Long runs are frequently conducted on undulating terrain and in group settings, producing natural variations in pacing and intensity. This variability may contribute to aerobic development, neuromuscular adaptation, and fatigue resistance (Casado et al., 2022; Haugen et al., 2022).

Additionally, the ability to sustain high training frequency in East African runners may be supported by gradual long-term development and early exposure to running-related activities. Previous studies have reported high levels of habitual physical activity, active transportation, and substantial daily energy expenditure among Kenyan youth, which may contribute to the development of aerobic capacity and training tolerance later in life (Onywera et al., 2006; Gibson et al., 2013; Ojiambo et al., 2013).

Group-based training represents a defining characteristic of East African endurance runners. Athletes typically train in large groups, often composed of runners of different performance levels, where pacing emerges dynamically rather than being externally prescribed (Wilber and Pitsiladis, 2012). This collective training environment promotes internally regulated intensity, competitive stimulus, and natural variability in training load (Billat et al., 2003; Casado et al., 2022).

Training groups frequently include progressive pacing structures, in which athletes gradually increase speed throughout the session. This pattern is commonly observed during fartlek workouts, tempo runs, and long runs performed on undulating terrain (Larsen, 2003). Such sessions allow athletes to self-regulate effort while responding to group dynamics, resulting in fluctuating intensities within the same training session (Wilber and Pitsiladis, 2012; Casado et al., 2022). The group-based structure may also enhance pacing behavior and fatigue tolerance. Athletes often adjust effort relative to other runners within the group, creating a competitive yet cooperative environment that promotes sustained training intensity without strict physiological monitoring (Santos-Concejero et al., 2015). This internally regulated pacing strategy contrasts with laboratory-based intensity prescription models commonly used in structured endurance training (Haugen et al., 2022).

Additionally, group training may facilitate higher training volume and consistency. Shared sessions improve adherence and provide psychological support during demanding workouts, which may contribute to long-term development and training sustainability in East African runners (Wilber and Pitsiladis, 2012; Casado et al., 2022).

### 3.2 Mixed-intensity sessions and implicit threshold exposure

Fartlek training represents a defining component of East African endurance training. These sessions are characterized by continuous running with repeated variations in pace and terrain (Foster et al., 2025). In practice, these sessions often incorporate elements of tempo running and interval-like surges, combining moderate-intensity aerobic running with intermittent efforts approaching LT or even vVO_2max_ speeds. This mixed-intensity structure allows athletes to accumulate substantial time at physiologically demanding intensities within a single workout (Billat et al., 2003; Wilber and Pitsiladis, 2012; Casado et al., 2022).

Evidence suggests that East African runners perform a greater proportion of training at high intensities compared with non-East African athletes, even when total training volume is similar. Coetzer et al. (1993) reported that East African runners accumulated more training above 80% VO_2max_, whereas low-intensity training volume did not differ between groups, indicating that intensity variability may represent a distinguishing characteristic of their training model. Billat et al. (2003) demonstrated that elite Kenyan runners frequently train either at velocities near LT or at vVO_2max_, using both continuous tempo runs and interval-based formats. Continuous tempo sessions lasting 45–70 min are commonly used, alongside longer aerobic intervals such as repeated mile efforts with short recovery periods, allowing athletes to gradually increase running speed throughout the session (Billat et al., 2003; Larsen, 2003; Casado et al., 2022). These workouts are often embedded within group-based fartlek sessions, where pace changes emerge naturally through collective dynamics rather than rigid prescriptions. This structure enables repeated surges within continuous running, exposing athletes to intensities near vLT and vVO_2max_ while maintaining overall aerobic volume (Wilber and Pitsiladis, 2012; Santos-Concejero et al., 2015).

Additionally, Kenyan runners frequently perform structured high-intensity repetitions within fartlek or progression-style workouts. Classic sessions include repeated 200 m intervals performed at approximately 120% of vVO_2max_ with long recovery periods, as well as 10–20 repetitions of 400–600 m at velocities close to vVO_2max_. Longer interval formats such as repeated mile efforts at intermediate speeds between 3,000 m and 10,000 m race pace are also commonly reported. These sessions further illustrate the variability-driven structure of East African training, combining aerobic running with intermittent high-intensity efforts within continuous workouts (Billat et al., 2003; Casado et al., 2022).

### 3.3 Training variability, hill training, and implicit threshold exposure

Hill running has been shown to improve running economy (RE) and neuromuscular efficiency in endurance runners, particularly through increased muscular recruitment and greater metabolic demand during uphill locomotion (Barnes et al., 2013; Barnes and Kilding, 2015). These adaptations may enhance fatigue resistance and performance during prolonged running.

Training on hilly terrain represents a common characteristic of East African endurance training environments. Kenyan, Ethiopian, and Ugandan runners frequently perform a large proportion of their training on undulating dirt roads and rolling terrain at moderate altitude (typically ~2,100–2,900 m), particularly in regions such as the Rift Valley and highland areas (Larsen, 2003; Wilber and Pitsiladis, 2012; Mooses and Hackney, 2017; Haugen et al., 2022; Grivas et al., 2024). Such terrain introduces continuous fluctuations in running speed and physiological load, which may promote aerobic development and pacing adaptability (Larsen, 2003; Wilber and Pitsiladis, 2012). Field observations of East African training groups further indicate that athletes often incorporate long climbs and variable terrain within daily runs rather than performing structured hill repetitions (Larsen, 2003; Haugen et al., 2022).

In addition, East African runners commonly train on unpaved roads and trails, which combine soft surfaces with frequent elevation changes. This environmental characteristic may increase neuromuscular demands while reducing repetitive impact forces, allowing athletes to tolerate high weekly training volumes and maintain long-term training consistency (Onywera et al., 2006; Mooses and Hackney, 2017).

Early exposure to rolling terrain may also contribute to strength development and RE adaptations. Many East African runners grow up in rural environments where daily locomotion involves walking or running over uneven terrain, which may further enhance musculoskeletal robustness and endurance performance (Saltin et al., 1995; Onywera et al., 2006).

Implicit threshold exposure appears to be a characteristic feature of East African endurance training. Rather than performing strictly controlled threshold sessions based on lactate or heart rate measurements, Kenyan, Ethiopian, and Ugandan runners frequently reach threshold intensities naturally during fartlek sessions, progression runs, and fast group-based aerobic runs (Wilber and Pitsiladis, 2012; Casado et al., 2022). These workouts often include sustained segments performed near maximal metabolic steady state without rigid pacing control, particularly during medium-long runs and continuous tempo efforts (Billat, 2001).

Observational reports from East African training camps indicate that moderate-to-fast continuous running is commonly performed on rolling terrain, where pace is internally regulated and influenced by group dynamics (Wilber and Pitsiladis, 2012). This structure results in prolonged exposure to intensities close to LT, especially during fartlek sessions and progression runs (Casado et al., 2022). Such implicit threshold exposure may enhance lactate clearance capacity, mitochondrial adaptations, and fatigue resistance, which are key determinants of endurance performance (Joyner and Coyle, 2008).

Physiological models of elite marathon performance also highlight the importance of sustained running near maximal lactate steady state (MLSS). Prolonged exposure to near-threshold intensity improves metabolic efficiency, substrate utilization, and fatigue resistance during long-duration exercise (Billat, 2001; Joyner and Coyle, 2008). Similar mechanisms have been proposed in models of sub-2-hour marathon performance, where maintaining intensities close to MLSS is considered critical for optimizing endurance performance and delaying fatigue (Grivas, 2026).

In addition, progression runs are frequently used in East African training groups, where athletes gradually increase pace during long aerobic sessions (Wilber and Pitsiladis, 2012). This structure naturally transitions from low intensity to threshold intensity within the same session, providing additional aerobic and neuromuscular stimuli without rigid intensity prescription (Seiler, 2010). Such training may contribute to the ability of East African runners to sustain fast race paces over long distances and tolerate prolonged high-intensity aerobic work (Casado et al., 2022; Grivas, 2026).

## 4 The Norwegian endurance training model

Despite its relatively small population, Norway has produced several world-class endurance runners across multiple generations, including Grete Waitz and Ingrid Kristiansen in the 1980s, Marius Bakken in the early 2000s, and more recently the Ingebrigtsen brothers and Narve Nordås, highlighting a consistent tradition of high-level performance (Kelemen et al., 2024). The training of these athletes has been systematically documented, revealing a relatively coherent training structure characterized by high training volume, controlled intensity distribution, and structured lactate-guided sessions (Enoksen et al., 2011; Stöggl and Sperlich, 2014; Kelemen et al., 2024; Sandbakk et al., 2025).

Endurance performance in middle- and long-distance running is primarily determined by physiological variables such as VO_2max_, RE, and vVO_2max_ (Conley and Krahenbuhl, 1980; Noakes et al., 1990; Grivas, 2020). In addition, the LT and associated running speed have been identified as strong predictors of endurance performance (Midgley et al., 2007; Faude et al., 2009). The interaction between training volume, intensity distribution, and training density plays a central role in the development of these characteristics, with different endurance models emphasizing distinct combinations of these variables (Midgley et al., 2007; Stöggl and Sperlich, 2014).

The Norwegian endurance training model is typically characterized by high volumes of low-intensity training combined with controlled moderate-intensity sessions regulated using physiological monitoring, and limited exposure to very high intensities (Enoksen et al., 2011; Stöggl and Sperlich, 2014; Kelemen et al., 2024; Sandbakk et al., 2025). Weekly running volume in elite Norwegian distance runners has been reported around ~160 km, with peaks reaching approximately 180–200 km during preparation phases and lower volumes during the competitive season (Kelemen et al., 2025; Sandbakk et al., 2025). Training intensity is commonly regulated using physiological monitoring, particularly blood lactate measurements, allowing athletes to maintain stable metabolic responses across sessions (Kelemen et al., 2024; Sandbakk et al., 2025). This controlled intensity approach enables athletes to accumulate substantial training volume at moderate physiological intensity while maintaining training quality and minimizing fatigue (Kelemen et al., 2024). Altitude exposure may also be incorporated strategically within the Norwegian endurance model, particularly during specific preparation phases, with session intensity and recovery adjusted to maintain the desired physiological stimulus while limiting excessive fatigue (Tønnessen et al., 2024).

### 4.1 Lactate-guided regulation, double-threshold training, and threshold density

A central characteristic of the Norwegian endurance training model is the systematic use of lactate-controlled training to regulate exercise intensity and optimize metabolic adaptations. Rather than prescribing intensity solely based on speed, heart rate, or perceived exertion, Norwegian athletes frequently monitor blood lactate concentration during training sessions to ensure that exercise intensity remains within predefined physiological zones (Enoksen et al., 2011; Kelemen et al., 2024). This approach allows athletes to accumulate substantial training volume near the LT while minimizing excessive physiological stress and maintaining training quality (Midgley et al., 2007; Faude et al., 2009; Vijay et al., 2024).

Typically, lactate-guided sessions in the Norwegian model are performed at blood lactate concentrations ranging approximately between 2–4 mmol·L^-^¹, corresponding to intensities near the LT (Jones and Carter, 2000; Faude et al., 2009). Maintaining lactate within this range enables athletes to perform prolonged or repeated moderate-intensity efforts without excessive fatigue, thereby increasing total training volume at controlled metabolic intensity (Midgley et al., 2007; Kelemen et al., 2024). This controlled metabolic approach differs from traditional interval-based high-intensity training, which often produces higher lactate accumulation and greater neuromuscular fatigue (Faude et al., 2009; Stöggl and Sperlich, 2014).

Another important aspect of lactate-controlled training is the stability of physiological responses across repetitions and sessions. Athletes aim to maintain relatively constant lactate values throughout intervals, adjusting running speed accordingly (Kelemen et al., 2024). This strategy allows precise control of internal load and reduces variability in metabolic stress, facilitating frequent threshold sessions within the weekly training structure (Enoksen et al., 2011; Kelemen et al., 2024). Such regulation supports the high frequency of threshold-based workouts commonly observed in Norwegian endurance athletes (Kelemen et al., 2024).

Overall, lactate-controlled training enables endurance athletes to combine high training volume with moderate-intensity metabolic stress, promoting improvements in aerobic capacity, lactate clearance, and endurance performance while limiting excessive fatigue accumulation (Midgley et al., 2007; Faude et al., 2009).

Double-threshold training has been described as a commonly used strategy within the Norwegian endurance training model, involving two lactate-guided moderate-intensity sessions performed within the same day (Kelemen et al., 2024). This approach has been documented in elite Norwegian middle- and long-distance runners, particularly in case studies of the Ingebrigtsen brothers, where sessions are completed both in the morning and afternoon to increase training frequency while maintaining controlled physiological responses (Tjelta, 2013, Tjelta, 2019; Kelemen et al., 2024). These sessions are typically performed at intensities near the LT, usually within a blood lactate range of approximately 2–4.5 mmol·L^-^¹, allowing athletes to accumulate substantial training volume at moderate metabolic stress (Seiler and Kjerland, 2006; Casado et al., 2023).

Morning sessions often consist of longer intervals, such as repeated 6-min efforts or 2–3 km intervals, whereas afternoon sessions include shorter intervals, such as 10–12 × 1000 m or 20–25 × 400 m with short recovery periods (Tjelta, 2013, Tjelta, 2019; Kelemen et al., 2024). The interval format may also enable athletes to run at relatively high absolute speeds despite moderate metabolic intensity, thereby increasing motor unit recruitment while preserving metabolic control (Casado et al., 2023). In practical implementations of the Norwegian model, morning sessions often include formats such as 5 × 6 min or 3 × 10 min performed at lactate concentrations typically below ~2.5 mmol·L^-^¹, whereas afternoon sessions consist of shorter intervals such as 10–12 × 1000 m, 25 × 400 m, or short repetitions (e.g., 45 s with brief recovery) (Kelemen et al., 2025).

Observational evidence suggests that double-threshold days are commonly performed twice per week during the preparation period, contributing to a weekly structure characterized by multiple moderate-intensity sessions, one VO_2max_-oriented session, and a high overall volume of low-intensity training (Casado et al., 2023; Kelemen et al., 2024). This distribution enables athletes to increase training volume at controlled metabolic intensity while limiting the amount of training performed at very high intensities.

A key component of the double-threshold approach is the use of blood lactate monitoring to regulate internal load. Lactate measurements are taken during the session to ensure that exercise intensity remains within the targeted metabolic range, reducing the likelihood of excessive central and peripheral fatigue and facilitating repeated moderate-intensity exposure across the training week (Casado et al., 2023; Kelemen et al., 2024). Although direct experimental evidence specifically examining double-threshold training remains limited, current descriptive evidence suggests that this approach represents a practical strategy for increasing the volume of work performed near the LT within a controlled physiological framework (Casado et al., 2023; Kelemen et al., 2024).Threshold density is another defining feature of the Norwegian endurance training model and refers to the large amount of training performed near the LT within the weekly microcycle. Rather than relying primarily on isolated high-intensity sessions, Norwegian runners typically accumulate a substantial volume of work at threshold intensity through repeated interval-based sessions distributed across the week (Casado et al., 2023; Kelemen et al., 2024).

Observational studies of elite Norwegian runners indicate that training at or near the LT typically accounts for approximately 15–25% of total training volume, although this proportion varies across training phases and may increase during preparation periods when double-threshold sessions are implemented (Tjelta and Enoksen, 2010; Enoksen et al., 2011; Kelemen et al., 2024). In the Ingebrigtsen model, threshold work is often performed two to four times per week, frequently organized as two double-threshold days, thereby increasing the total amount of work performed at intensities close to the LT (Tjelta, 2019; Kelemen et al., 2024). This distribution suggests that structured moderate-intensity sessions represent an important component of the Norwegian training structure, particularly during preparation periods. In specific preparation phases, this organization may allow athletes to accumulate substantial volumes of threshold training, with double-threshold days in some cases reaching approximately 20–24 km and weekly threshold-training volumes becoming relatively high. However, such threshold work remains embedded within an overall intensity distribution dominated by low-intensity training, consistent with polarized or pyramidal models described in Norwegian endurance athletes (Kelemen et al., 2025).

The physiological rationale for high threshold density lies in the possibility of accumulating a large amount of race-relevant aerobic work while maintaining controlled internal load. Training near the second lactate threshold (LT2) has been associated with improvements in velocity at threshold, lactate clearance, aerobic power, and endurance performance (Midgley et al., 2007; Faude et al., 2009; Casado et al., 2023). When such work is distributed across multiple sessions, athletes may tolerate a greater weekly volume of threshold training than would be possible if the same amount of work were concentrated in fewer, more fatiguing sessions (Casado et al., 2023; Kelemen et al., 2024).

An important practical consequence of this approach is that it enables a high frequency of structured moderate-intensity sessions without a corresponding increase in very high-intensity training. In the Norwegian model, the preparation period is typically characterized by a high proportion of low-intensity training, complemented by regular lactate-guided moderate-intensity sessions, while exposure to very high intensities remains relatively limited (Tjelta, 2013, Tjelta, 2016; Kelemen et al., 2024). This strategy may help explain how Norwegian runners are able to maintain both high training consistency and high physiological specificity throughout the season.

Overall, threshold density appears to represent a key mechanism through which the Norwegian endurance training model combines high-volume training with precise metabolic control. By increasing the weekly amount of work performed near the LT, athletes may optimize aerobic adaptation while minimizing excessive fatigue and preserving long-term training quality (Casado et al., 2023; Kelemen et al., 2024).

### 4.2 Structured intervals and selective high-intensity exposure

Structured interval sessions are widely used in the Norwegian endurance training model to organize threshold training in a controlled and repeatable manner. Instead of continuous threshold runs, athletes typically perform repeated intervals with short recovery periods, allowing consistent pacing and stable physiological responses across the session (Tjelta, 2013; Casado et al., 2023; Kelemen et al., 2024).

Reported session formats include moderate-duration intervals such as 5–6 × 6 min, 6–8 × 1000 m, and 10–12 × 1000 m, as well as shorter intervals such as 20–25 × 400 m. Recovery periods are usually brief, commonly ranging from 30 to 60 s, helping preserve a stable internal load across repetitions (Tjelta, 2013, Tjelta, 2019; Kelemen et al., 2024). The interval-based structure allows partial recovery between repetitions, facilitating a balance between training intensity and total work performed. This format may allow athletes to accumulate substantial aerobic work while maintaining tighter control of fatigue than during continuous threshold efforts. In addition, the repeated nature of the intervals allows adjustments in pacing throughout the session, supporting consistent execution across repetitions (Casado et al., 2023). Structured interval sessions therefore offer a practical and repeatable approach to organizing moderate-intensity training within the Norwegian model (Casado et al., 2023; Kelemen et al., 2024).

Limited use of high-intensity training is another characteristic of the Norwegian endurance training model. While high-intensity interval training is part of endurance training practice, Norwegian athletes generally use it sparingly, particularly during the general preparation period (Seiler and Kjerland, 2006; Tjelta, 2019; Casado et al., 2023).

Observational descriptions of elite Norwegian runners indicate that a substantial proportion of structured sessions are performed near the LT, whereas training performed well above threshold is relatively infrequent. In many cases, only one high-intensity session per week is included, often consisting of VO_2max_-oriented intervals such as 4–6 × 3–5 min or similar formats (Tjelta, 2013; Kelemen et al., 2024). Accordingly, the Norwegian model appears to emphasize controlled moderate-intensity exposure, with high-intensity work used more selectively.

Limiting the amount of high-intensity training may help reduce neuromuscular and metabolic strain, thereby supporting training consistency and tolerance to higher weekly training volumes. High-intensity sessions are typically placed strategically within the weekly structure, often separated by low-intensity training days, to preserve recovery and avoid excessive fatigue accumulation (Seiler and Kjerland, 2006; Casado et al., 2023).

This restrained use of high-intensity training also aligns with the broader emphasis on stable physiological responses and sustainable training loads. Rather than relying on repeated exposure to maximal intensities, the Norwegian model is primarily characterized by high-volume low-intensity running, complemented by structured, lactate-guided moderate-intensity sessions. High-intensity sessions are therefore used selectively to complement aerobic development rather than to serve as the dominant training stimulus (Casado et al., 2023; Kelemen et al., 2024).

Overall, the limited use of high-intensity training appears to support a sustainable balance between training stimulus and recovery within the Norwegian endurance training model.

## 5 Training organization comparison

While both East African and Norwegian training systems share several common principles, including high training volume and frequent submaximal sessions, important differences emerge in how training is organized and executed. These differences relate primarily to variability, intensity regulation, pacing structure, and degree of individualization. The following sections compare these models across key organizational dimensions, including variability versus stability, implicit versus explicit threshold regulation, and group-based versus individualized training.

One of the primary differences between East African and Norwegian endurance training systems lies in the degree of variability in training organization. Observational studies of East African runners consistently describe training characterized by fluctuating pacing, mixed-intensity sessions, and flexible execution within group-based workouts. Early field observations of Kenyan athletes reported fartlek-style training performed on undulating terrain, where pace changes emerged naturally rather than being externally prescribed (Billat et al., 2003). Similar descriptions emphasized that group dynamics frequently determine intensity changes, producing variable physiological demands across sessions (Larsen, 2003). Subsequent analyses confirmed that East African runners often combine continuous running with surges and progression efforts within the same session, resulting in substantial within-session variability and flexible intensity distribution (Wilber and Pitsiladis, 2012). These characteristics suggest a training organization in which pacing and intensity are adjusted dynamically rather than strictly controlled.

In contrast, the Norwegian endurance training model is characterized by greater stability in training organization and intensity execution. Early descriptive studies of Norwegian middle- and long-distance runners reported structured interval-based sessions designed to maintain consistent pacing and physiological responses across repetitions (Tjelta and Enoksen, 2010). A longitudinal case study of the 2012 European 1500 m champion further demonstrated repeatable moderate-intensity sessions performed with controlled pacing and limited variability between intervals (Tjelta, 2013). Similar characteristics were later described in elite Norwegian training systems, including high training volume combined with systematically organized threshold sessions and consistent internal load regulation (Tjelta, 2019).

Overall, East African training appears to involve greater variability in pacing and session execution, whereas the Norwegian model places greater emphasis on stability and repeatable session structure. This interpretation is supported by evidence that both East African and Norwegian endurance systems have produced world-class performers despite relying on different approaches to training organization, intensity regulation, pacing structure, and physiological monitoring (Wilber and Pitsiladis, 2012; Sandbakk and Holmberg, 2017; Haugen et al., 2022; Kelemen et al., 2024).

Another distinction between East African and Norwegian endurance training systems concerns how threshold intensity is regulated. In East African training environments, threshold intensity is typically controlled implicitly through perceived effort, group dynamics, and terrain. Observational descriptions of Kenyan runners indicate that tempo runs, fartlek sessions, and progression workouts are commonly performed without strict physiological monitoring, with athletes adjusting pace dynamically within the group (Billat, 2001; Larsen, 2003; Wilber and Pitsiladis, 2012; Grivas et al., 2024). As a result, athletes frequently operate near threshold intensity, but without explicit measurement or rigid control.

In contrast, the Norwegian model relies on explicit regulation of threshold intensity. Studies describing Norwegian runners emphasize structured sessions designed to maintain consistent physiological responses and controlled internal load (Tjelta and Enoksen, 2010; Tønnessen et al., 2014; Sandbakk and Holmberg, 2017; Sandbakk et al., 2025). Recent descriptions of this approach also highlight the systematic regulation of intensity during lactate-guided sessions to promote consistent internal responses across repeated training exposures (Haugen et al., 2022; Casado et al., 2023).

Thus, East African runners tend to regulate threshold intensity implicitly through self-selected pacing, whereas Norwegian athletes employ explicit physiological control. These contrasting approaches suggest that threshold-related adaptations may be achieved either through adaptive self-regulation or through structured physiological control.

East African endurance training is commonly performed in large groups, with pacing often emerging through pack dynamics rather than strictly individualized prescription. Observational studies of Kenyan training environments describe athletes completing fartlek sessions, tempo runs, and interval-based workouts collectively, with pace adjustments shaped by group behavior (Billat et al., 2003; Larsen, 2003). Field-based reports further note that athletes frequently train in large packs, within which progressive pacing and competitive surges arise naturally during the session (Onywera et al., 2006; Wilber and Pitsiladis, 2012). Analyses of East African training characteristics also highlight shared session formats, group-based long runs, and collective execution of key workouts, suggesting that training intensity is frequently regulated through social dynamics rather than individualized targets (Saltin et al., 1995; Faude et al., 2009; Haugen et al., 2022).

In contrast, Norwegian endurance training environments generally allow greater individual regulation of intensity within shared session structures. Descriptive studies of Norwegian runners report structured interval sessions designed to maintain consistent physiological responses, with pacing adjusted according to individual internal load rather than a uniform group speed (Tjelta and Enoksen, 2010). A longitudinal case study of elite Norwegian middle-distance preparation described repeatable lactate-guided sessions in which athletes completed identical workout formats while regulating intensity individually (Tjelta, 2013). More recent descriptions of lactate-guided threshold training likewise indicate that athletes regulate intensity individually while participating in common session structures (Casado et al., 2023; Kelemen et al., 2024). In addition, best-practice accounts from Norwegian world-class coaches emphasize systematic monitoring, individualized adjustment, and continuous coach–athlete dialogue to optimize training load and session execution (Sandbakk et al., 2025).

Overall, East African training environments appear to emphasize collective execution and group-mediated pacing, whereas Norwegian systems place greater emphasis on individualized intensity regulation within structured workouts. A conceptual comparison of the main organizational and physiological characteristics of the East African and Norwegian endurance training systems is presented in **Table 1**.

**Table 1:** Conceptual comparison of selected characteristics of East African and Norwegian endurance training systems.

Feature	East African model	Norwegian model
Training organization	Variability-driven, group-based	Structured, physiologically regulated
Intensity regulation	Mostly implicit and internally regulated	Explicit and physiologically regulated
Session characteristics	Fartlek, progressive runs, group training	Structured threshold intervals
Pacing characteristics	Variable, surge-based	Controlled, repeatable
Threshold exposure	Often implicit within mixed sessions	Explicit and systematically organized
Lactate monitoring	Rare	Frequent
Altitude context	Altitude-associated living/training environment	Strategic altitude training/preparation
Primary physiological emphasis	Adaptability, recruitment diversity	Metabolic efficiency, lactate regulation
Neuromuscular profile	Variable demands	More controlled demands
Durability-related emphasis	Robustness under variable workloads	Sustained submaximal efficiency
Race-preparation relevance	Tactical variability and response to surges	Steady-state control and sustained pace
Overall training orientation	Ecologically regulated	Physiologically regulated

## 6 Physiological adaptation pathways

Different endurance training organizations may promote distinct physiological adaptation pathways leading to elite performance. The Norwegian threshold-density model emphasizes frequent exposure to intensities near the LT, which may enhance oxidative metabolism and improve fractional utilization of VO_2max_ (Seiler, 2010; Sandbakk and Holmberg, 2017). Training structures observed in elite Norwegian runners indicate a strong emphasis on work performed near the LT, with lactate-guided regulation helping to standardize internal load across sessions (Tjelta and Enoksen, 2010).

Recent descriptions of Norwegian endurance coaching practices further highlight interval sessions characterized by high accumulated volume and controlled execution, which may facilitate repeated exposure to submaximal workloads and improve aerobic adaptations (Tønnessen et al., 2024). Such training organization may enhance mitochondrial biogenesis, improve lactate clearance capacity, and increase efficiency during prolonged exercise. Furthermore, structured threshold work has been associated with improved durability, defined as the ability to maintain physiological efficiency during long-duration efforts (Haugen et al., 2022; Kelemen et al., 2024). These adaptations may allow athletes to sustain high aerobic output while minimizing excessive neuromuscular fatigue.

In contrast, variability-driven training commonly observed in East African runners may promote physiological adaptations through fluctuating intensity patterns and mixed pacing stimuli. Observational studies of Kenyan runners report frequent changes in running speed, fartlek sessions, and progressive long runs, exposing athletes to variable metabolic demands (Saltin et al., 1995; Onywera et al., 2006; Wilber and Pitsiladis, 2012). Such variability may increase recruitment diversity, enhance neuromuscular adaptability, and improve tolerance to pace fluctuations during prolonged running (Billat, 2001; Casado et al., 2023). Recent evidence from elite Ethiopian runners further supports the variability-driven adaptation pathway. Worku et al. (2026) reported that sequential blocks of endurance, strength, and speed training were associated with complementary physiological adaptations and improved 5000 m performance in altitude-adapted Ethiopian distance runners. This mixed-modality structure may enhance metabolic flexibility, RE, and fatigue resistance, consistent with variability-driven training organization observed in East African endurance systems. Repeated exposure to variable intensities may also broaden the range of physiological stressors and improve performance under dynamically changing race conditions (Wilber and Pitsiladis, 2012; Haugen et al., 2022; Casado et al., 2023).

Together, these findings suggest that endurance performance may develop through at least two complementary physiological pathways. Threshold-density training may primarily enhance metabolic efficiency, lactate dynamics, and steady-state durability, whereas variability-driven training may emphasize adaptability, recruitment variability, and tolerance to pacing fluctuations.

## 7 Converging pathways to elite endurance performance

The comparison between Norwegian threshold-density training and East African variability-driven training suggests that elite endurance performance may emerge through different but complementary pathways. Despite clear differences in training organization, pacing regulation, and session structure, both training systems appear capable of producing world-class endurance athletes (Seiler, 2010; Wilber and Pitsiladis, 2012; Sandbakk and Holmberg, 2017; Casado et al., 2022).

Importantly, these pathways should not be interpreted as mutually exclusive. Both models incorporate substantial low-intensity training and periodic exposure to higher intensities, suggesting that performance development likely depends on the interaction between training volume, intensity distribution, and physiological regulation. Taken together, these observations support the view that elite endurance performance can arise from different training organizations without requiring a single dominant model. This perspective may provide a broader conceptual framework for interpreting successful endurance systems and for designing training approaches adapted to the athlete, context, and competitive demands.

## 8 Practical applications

The comparison between threshold-density and variability-driven training systems suggests that endurance training practice may benefit from considering how different session organizations target different performance-related characteristics. Structured threshold sessions may support metabolic efficiency and lactate regulation, whereas more variable session formats may enhance adaptability, tolerance to pace fluctuations, and fatigue resistance during prolonged exercise. From a practical perspective, coaches may draw on selected features of both systems according to athlete needs, event demands, and training phase. In applied settings, controlled moderate-intensity intervals may be used to develop sustainable aerobic output near the LT, whereas variability-based sessions may include progressive runs, fartlek training, or mixed-intensity long runs that expose athletes to more dynamic physiological demands. The relative emphasis placed on these elements may vary according to competition schedule, seasonal periodization, and individual athlete response. This perspective may also support individualized training strategies. Athletes with strong aerobic capacity but limited fatigue resistance may benefit from greater exposure to training formats that incorporate pace variation and mixed-intensity demands, whereas athletes requiring improved metabolic efficiency may benefit from greater emphasis on lactate-guided moderate-intensity work. In addition, lactate-guided moderate-intensity training may improve the ability to sustain a high fraction of maximal aerobic capacity, whereas variability-based sessions may better prepare athletes to respond to surges, tactical changes, and uneven pacing commonly observed in competition. Overall, the present comparison provides a practical framework for interpreting how different training organizations may contribute to endurance performance. Rather than prescribing a fixed combined model, it highlights how selected elements from threshold-density and variability-driven systems may inform context-specific and individualized training practice.

## 9 Limitations

This narrative review has several limitations that should be considered when interpreting the conceptual framework presented in this paper. First, the comparison between Norwegian threshold-density and East African variability-driven training models is primarily based on observational and descriptive studies rather than controlled experimental designs. As a result, causal relationships between training organization and physiological adaptations cannot be definitively established. The available literature often relies on retrospective analyses, training diaries, and observational reports, which may introduce methodological variability and limit generalizability. Second, endurance performance is influenced by multiple factors beyond training structure, including genetic predisposition, environmental conditions, altitude exposure, socioeconomic background, and long-term athlete development pathways. East African success, for example, has been associated not only with training characteristics but also with altitude living, active lifestyles, and cultural factors. Similarly, Norwegian training systems may reflect strong coaching infrastructure, monitoring practices, and individualized training approaches. These factors may interact with training organization and contribute to performance outcomes. Third, the present framework is conceptual and has not been directly tested in controlled longitudinal studies. Although it is informed by indirect evidence from different training systems, experimental research is still needed to clarify how variability-driven and threshold-density training characteristics influence physiological adaptations, durability, and endurance performance across different athlete populations. Finally, individual responses to training vary substantially, and the relative effectiveness of threshold-density versus variability-driven approaches may depend on athlete characteristics, training history, and competition demands. Therefore, the present framework should be interpreted as a conceptual tool rather than as a prescriptive training template. These limitations highlight the need for future research examining how different endurance training organizations influence physiological adaptation and performance.

## 10 Conclusion

Elite endurance performance may emerge through multiple training pathways characterized by different organizational principles and physiological adaptations. Variability-driven training, commonly observed in East African runners, appears to promote adaptability, fatigue resistance, and tolerance to pace fluctuations, whereas threshold-density training, frequently reported in Norwegian endurance systems, may enhance metabolic efficiency, stable physiological responses, and submaximal durability. Despite these differences, both approaches appear capable of producing world-class endurance athletes, suggesting that endurance performance is not dependent on a single optimal training model. The comparison of these training systems indicates that different training organizations may target partially distinct yet complementary determinants of endurance performance. Variability-driven training may improve responsiveness to stochastic race dynamics, while threshold-density training may support sustained aerobic output and metabolic stability. Rather than supporting a single prescriptive model, the present review provides a conceptual framework for understanding how different endurance training systems may contribute to elite performance across different athlete profiles, competition demands, and training environments. Overall, the framework presented in this review highlights that elite endurance development may be achieved through more than one organizational route. This perspective may help inform future research and may also offer practical value for coaches seeking to interpret and apply endurance training principles in context-specific ways.

## References

[B1] Barnes K. R. Hopkins W. G. McGuigan M. R. Kilding A. E. (2013). Effects of different uphill interval-training programs on running economy and performance. Int. J. Sport. Physiol. Perform. 8, 639–647. doi: 10.1123/ijspp.8.6.639 23538293

[B2] Barnes K. R. Kilding A. E. (2015). Running economy: measurement, norms, and determining factors. Sport. Med. - Open 1, 8. doi: 10.1186/s40798-015-0007-y 27747844 PMC4555089

[B3] Billat L. V. (2001). Interval training for performance: a scientific and empirical practice. Special recommendations for middle- and long-distance running. Part I: aerobic interval training. Sport. Med. 31, 13–31. doi: 10.2165/00007256-200131010-00002 11219499

[B4] Billat V. Lepretre P.-M. Heugas A.-M. Laurence M.-H. Salim D. Koralsztein J. P. (2003). Training and bioenergetic characteristics in elite male and female Kenyan runners. Med. Sci. Sport. Exerc. 35, 297–304. doi: 10.1249/01.MSS.0000053556.59992.A9 12569219

[B5] Casado A. Foster C. Bakken M. Tjelta L. I. (2023). Does lactate-guided threshold interval training within a high-volume low-intensity approach represent the “next step” in the evolution of distance running training? Int. J. Environ. Res. Public Health 20, 3782. doi: 10.3390/ijerph20053782 36900796 PMC10000870

[B6] Casado A. González-Mohíno F. González-Ravé J. M. Foster C. (2022). Training periodization, methods, intensity distribution, and volume in highly trained and elite distance runners: a systematic review. Int. J. Sport. Physiol. Perform. 17, 820–833. doi: 10.1123/ijspp.2021-0435 35418513

[B7] Coetzer P. Noakes T. D. Sanders B. Lambert M. I. Bosch A. N. Wiggins T. . (1993). Superior fatigue resistance of elite black South African distance runners. J. Appl. Physiol. 75, 1822–1827. doi: 10.1152/jappl.1993.75.4.1822 8282637

[B8] Conley D. L. Krahenbuhl G. S. (1980). Running economy and distance running performance of highly trained athletes. Med. Sci. Sport. Exerc. 12, 357–360. doi: 10.1249/00005768-198025000-00010 7453514

[B9] Enoksen E. Tjelta A. R. Tjelta L. I. (2011). Distribution of training volume and intensity of elite male and female track and marathon runners. Int. J. Sport. Sci. Coach. 6, 273–293. doi: 10.1260/1747-9541.6.2.273 39154575

[B10] Faude O. Kindermann W. Meyer T. (2009). Lactate threshold concepts: how valid are they? Sport. Med. 39, 469–490. doi: 10.2165/00007256-200939060-00003 19453206

[B11] Foster C. Casado A. Bok D. Hofmann P. Bakken M. Tjelta A. . (2025). History and perspectives on interval training in sport, health, and disease. Appl. Physiol. Nutr. Metab. Physiol. Appl. Nutr. Metab. 50, 1–16. doi: 10.1139/apnm-2023-0611 40272275

[B12] Gibson A. R. Ojiambo R. Konstabel K. Lieberman D. E. Reilly J. J. Speakman J. R. . (2013). Aerobic capacity, activity levels and daily energy expenditure in male and female adolescents of the Kenyan Nandi sub-group. PloS One 8, e66552. doi: 10.1371/journal.pone.0066552 23805234 PMC3689839

[B13] Grivas G. (2020). Physiological predictors of distance runners’ performance: a narrative review. Trends Sport. Sci. 27, 117–123. doi: 10.23829/TSS.2020.27.3-1

[B14] Grivas G. V. (2026). Toward a record-eligible sub-2-hour marathon: an updated integrative framework of physiological, technological, and cognitive determinants. Eur. J. Appl. Physiol. 126, 37–59. doi: 10.1007/s00421-025-06085-6 41351755

[B15] Grivas G. V. Onywera V. O. Marco-Contreras L. A. Sutehall S. Muniz-Pardos B. (2024). Why the dominance of East Africans in distance running? A narrative review. Transl. Exerc. Biomed. 1, 124–134. doi: 10.1515/teb-2024-0018 31755547

[B16] Haugen T. Sandbakk Ø. Seiler S. Tønnessen E. (2022). The training characteristics of world-class distance runners: an integration of scientific literature and results-proven practice. Sport. Med. - Open 8, 46. doi: 10.1186/s40798-022-00438-7 35362850 PMC8975965

[B17] Jones A. M. Carter H. (2000). The effect of endurance training on parameters of aerobic fitness. Sport. Med. 29, 373–386. doi: 10.2165/00007256-200029060-00001 10870864

[B18] Joyner M. J. Coyle E. F. (2008). Endurance exercise performance: the physiology of champions. J. Physiol. 586, 35–44. doi: 10.1113/jphysiol.2007.143834 17901124 PMC2375555

[B19] Kelemen B. Benczenleitner O. Tóth L. (2024). The Norwegian double-threshold method in distance running: systematic literature review. Sci. J. Sport. Perform. 3, 38–46. doi: 10.55860/NBXV4075

[B20] Kelemen B. Benczenleitner O. Tóth L. (2025). Emerging trends in distance running training: bridging science and empirical insights — a narrative review. Int. J. Sport. Sci. Coach. 20, 2779–2795. doi: 10.1177/17479541251356570

[B21] Larsen H. B. (2003). Kenyan dominance in distance running. Comp. Biochem. Physiol. A. Mol. Integr. Physiol. 136, 161–170. doi: 10.1016/s1095-6433(03)00227-7 14527638

[B22] McKay A. K. A. Stellingwerff T. Smith E. S. Martin D. T. Mujika I. Goosey-Tolfrey V. L. . (2022). Defining training and performance caliber: a participant classification framework. Int. J. Sport. Physiol. Perform. 17, 317–331. doi: 10.1123/ijspp.2021-0451 34965513

[B23] Midgley A. W. McNaughton L. R. Jones A. M. (2007). Training to enhance the physiological determinants of long-distance running performance: can valid recommendations be given to runners and coaches based on current scientific knowledge? Sport. Med. 37, 857–880. doi: 10.2165/00007256-200737100-00003 17887811

[B24] Mooses M. Hackney A. C. (2017). Anthropometrics and body composition in East African runners: potential impact on performance. Int. J. Sport. Physiol. Perform. 12, 422–430. doi: 10.1123/ijspp.2016-0408 27631418

[B25] Noakes T. D. Myburgh K. H. Schall R. (1990). Peak treadmill running velocity during the VO2 max test predicts running performance. J. Sport. Sci. 8, 35–45. doi: 10.1080/02640419008732129 2359150

[B26] Ojiambo R. Gibson A. R. Konstabel K. Lieberman D. E. Speakman J. R. Reilly J. J. . (2013). Free-living physical activity and energy expenditure of rural children and adolescents in the Nandi region of Kenya. Ann. Hum. Biol. 40, 318–323. doi: 10.3109/03014460.2013.775344 23837829

[B27] Onywera V. O. Scott R. A. Boit M. K. Pitsiladis Y. P. (2006). Demographic characteristics of elite Kenyan endurance runners. J. Sport. Sci. 24, 415–422. doi: 10.1080/02640410500189033 16492605

[B28] Saltin B. Larsen H. Terrados N. Bangsbo J. Bak T. Kim C. K. . (1995). Aerobic exercise capacity at sea level and at altitude in Kenyan boys, junior and senior runners compared with Scandinavian runners. Scand. J. Med. Sci. Sport. 5, 209–221. doi: 10.1111/j.1600-0838.1995.tb00037.x 7552766

[B29] Sandbakk Ø. Holmberg H.-C. (2017). Physiological capacity and training routines of elite cross-country skiers: approaching the upper limits of human endurance. Int. J. Sport. Physiol. Perform. 12, 1003–1011. doi: 10.1123/ijspp.2016-0749 28095083

[B30] Sandbakk Ø. Tønnessen E. Sandbakk S. B. Losnegard T. Seiler S. Haugen T. (2025). Best-practice training characteristics within Olympic endurance sports as described by Norwegian world-class coaches. Sport. Med. - Open 11, 45. doi: 10.1186/s40798-025-00848-3 40278987 PMC12031707

[B31] Santos-Concejero J. Billaut F. Grobler L. Oliván J. Noakes T. D. Tucker R. (2015). Maintained cerebral oxygenation during maximal self-paced exercise in elite Kenyan runners. J. Appl. Physiol. 118, 156–162. doi: 10.1152/japplphysiol.00909.2014 25414248

[B32] Seiler K. S. Kjerland G.Ø. (2006). Quantifying training intensity distribution in elite endurance athletes: is there evidence for an “optimal” distribution? Scand. J. Med. Sci. Sport. 16, 49–56. doi: 10.1111/j.1600-0838.2004.00418.x 16430681

[B33] Seiler S. (2010). What is best practice for training intensity and duration distribution in endurance athletes? Int. J. Sport. Physiol. Perform. 5, 276–291. doi: 10.1123/ijspp.5.3.276 20861519

[B34] Stöggl T. Sperlich B. (2014). Polarized training has greater impact on key endurance variables than threshold, high intensity, or high volume training. Front. Physiol. 5. doi: 10.3389/fphys.2014.00033 24550842 PMC3912323

[B35] Stöggl T. L. Sperlich B. (2015). The training intensity distribution among well-trained and elite endurance athletes. Front. Physiol. 6, 295. doi: 10.3389/fphys.2015.00295 26578968 PMC4621419

[B36] Sun Q. Yu Y. Cui J. Lin S. Wang X. Zhou T. (2025). Recent advances in training intensity distribution theory for cyclic endurance sports: theoretical foundations, model comparisons, and periodization characteristics. Front. Physiol. 16. doi: 10.3389/fphys.2025.1657892 41169886 PMC12568352

[B37] Tjelta L. I. (2013). A longitudinal case study of the training of the 2012 European 1500 m track champion. Int. J. Appl. Sport. Sci. 25, 11–18. doi: 10.24985/ijass.2013.25.1.11

[B38] Tjelta L. I. (2016). The training of international level distance runners. Int. J. Sport. Sci. Coach. 11, 122–134. doi: 10.1177/1747954115624813

[B39] Tjelta L. I. (2019). Three Norwegian brothers all European 1500 m champions: what is the secret? Int. J. Sport. Sci. Coach. 14, 694–700. doi: 10.1177/1747954119872321

[B40] Tjelta L. I. Enoksen E. (2010). Training characteristics of male junior cross country and track runners on European top level. Int. J. Sport. Sci. Coach. 5, 193–203. doi: 10.1260/1747-9541.5.2.193 39154575

[B41] Tønnessen E. Sandbakk Ø. Sandbakk S. B. Seiler S. Haugen T. (2024). Training session models in endurance sports: a Norwegian perspective on best practice recommendations. Sport. Med. 54, 2935–2953. doi: 10.1007/s40279-024-02067-4 39012575 PMC11560996

[B42] Tønnessen E. Sylta Ø. Haugen T. A. Hem E. Svendsen I. S. Seiler S. (2014). The road to gold: training and peaking characteristics in the year prior to a gold medal endurance performance. PloS One 9, e101796. doi: 10.1371/journal.pone.0101796 25019608 PMC4096917

[B43] Vijay S. A. Sivakumar C. Kumar P. V. Muralidharan C. K. Rajkumar K. V. Kannan K. R. . (2024). Lactate threshold training to improve long-distance running performance: a narrative review. Montenegrin. J. Sport. Sci. Med. 13, 19–29. doi: 10.26773/mjssm.240303

[B44] Wilber R. L. Pitsiladis Y. P. (2012). Kenyan and Ethiopian distance runners: what makes them so good? Int. J. Sport. Physiol. Perform. 7, 92–102. doi: 10.1123/ijspp.7.2.92 22634972

[B45] Worku N. Tola Z. B. Taddese A. (2026). Effects of training modalities on physiology, hematology, and performance in elite Ethiopian distance male athletes. BMC Res. Notes. 19, 223. doi: 10.1186/s13104-026-07798-3 41935338 PMC13181890

